# Primary breast peripheral T-cell lymphoma not otherwise specified: report of a case

**DOI:** 10.1007/s00595-013-0808-x

**Published:** 2014-01-07

**Authors:** Daisuke Muroya, Uhi Toh, Nobutaka Iwakuma, Shino Nakagawa, Mai Mishima, Ryuji Takahashi, Miki Takenaka, Kazuo Shirouzu, Yoshito Agaki

**Affiliations:** Department of Surgery, University of Kurume Faculty of Medicine, 67 Asahi-machi, Kurume, Fukuoka Japan

**Keywords:** Primary breast lymphoma, Peripheral T-cell lymphoma not otherwise specified (PTCL-NOS)

## Abstract

Malignant lymphomas of the breast are rare and primary breast lymphoma comprises <0.5 % of breast malignancies, within which T-cell lymphomas are an even rarer subset. We report a case of primary breast peripheral T-cell lymphoma not otherwise specified (PTCL-NOS). Histology of the biopsied specimen revealed CD2(+), CD3(+), CD4(+), CD5(−), CD7(+), CD8(−), CD20(−), CD25(−), CD30(+), CD56(−), bcl-2(−), EBV-ISH(−), TIA-I(−), and ATLA negative. The patient was treated with six cycles of the CHOP regimen and died 17 months after the diagnosis was made, despite complete remission after conventional chemotherapy. To our knowledge, only 18 cases of primary peripheral T-cell lymphoma of the breast and just one previous case of primary PTCL-NOS of the breast have been reported in Japan.

## Introduction

Primary breast lymphomas (PBLs) account for only 0.04–0.5 % of breast malignancies. The most frequent PBLs are of the B-cell type, whereas the T-cell type is especially rare. We report a case of primary breast peripheral T-cell lymphoma not otherwise specified (PTCL-NOS) and compare its features with those of the 17 cases of PBL reported in Japan. Low-grade lymphomas should be managed with excision biopsy and/or local radiation therapy, whereas high-grade lymphomas are generally managed with a systemic chemotherapy regimen combined with radiation therapy. Radical surgery is not recommended for breast PBL patients, as they are usually suffering from systemic disease [1–3]. This case report describes a patient with T-cell lymphoma involving the breast parenchyma.

## Case report

**Fig. 1:**
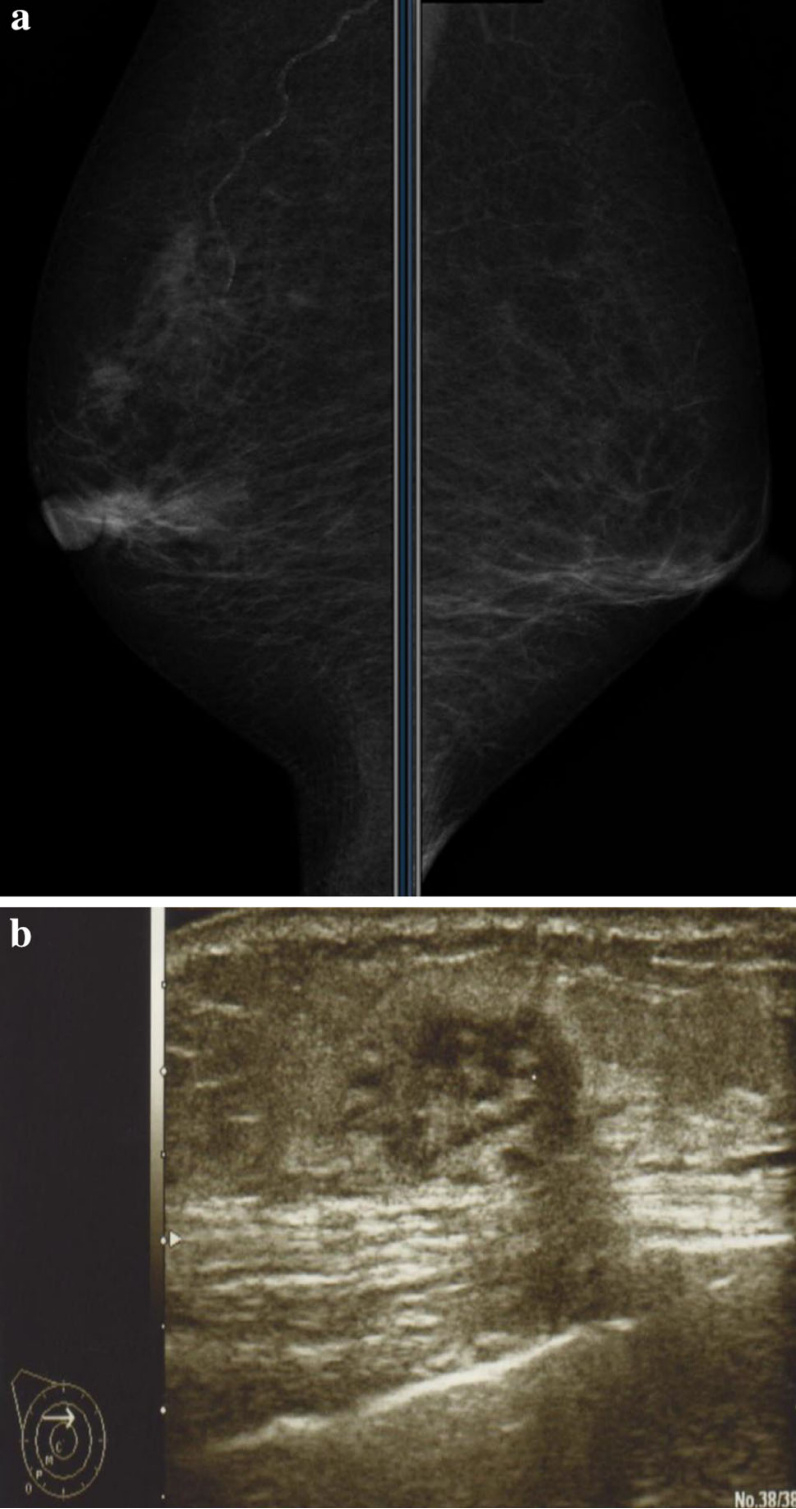
**a** Mammogram findings. The craniocaudal and mediolateral oblique (MLO) view revealed no mass lesion or other abnormality in the bilateral breasts. **b** Ultrasonography findings. An irregular, round and solid hypoechoic nodule in the 11:00 o’clock position corresponds to a nodule palpated on clinical examination

**Fig. 2:**
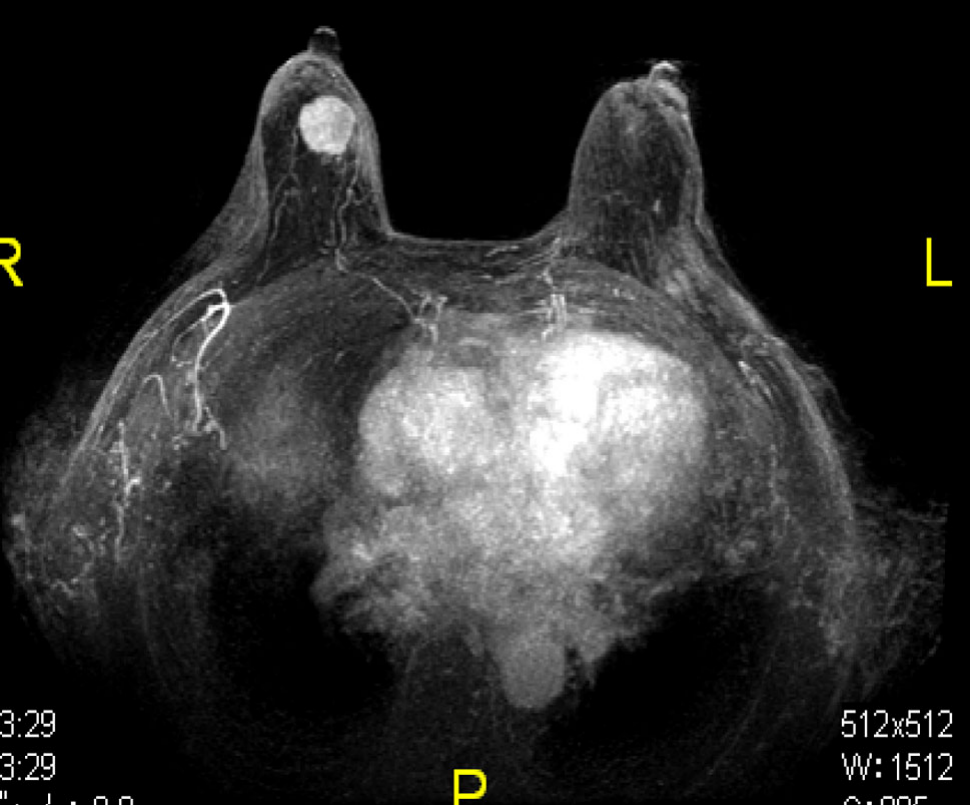
Magnetic resonance imaging findings. A 3 × 2 × 2 cm mass in the *upper right* breast showed a malignant imaging pattern on dynamic study

**Fig. 3:**
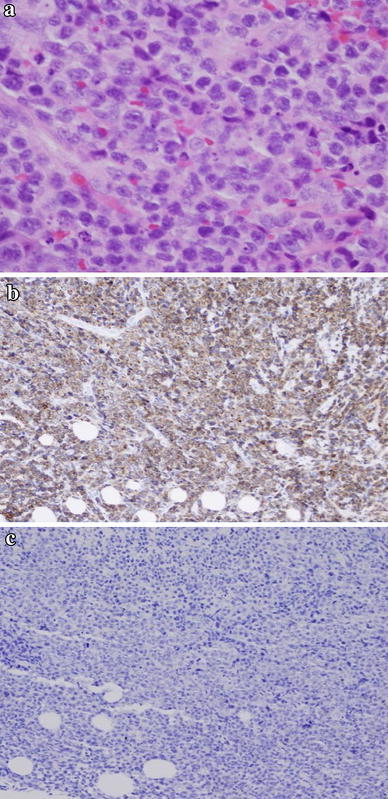
**a** Microscopic examination revealed neoplastic infiltrates composed of medium to large cells with round nuclei (HE, original magnification ×400). **b** Immunohistochemical stain of CD3 revealed a strongly positive reaction within the tumor tissue (original magnification ×400). **c** Immunohistochemical stain of CD20 revealed a negative reaction within the tumor tissue (original magnification ×100)

**Fig. 4:**
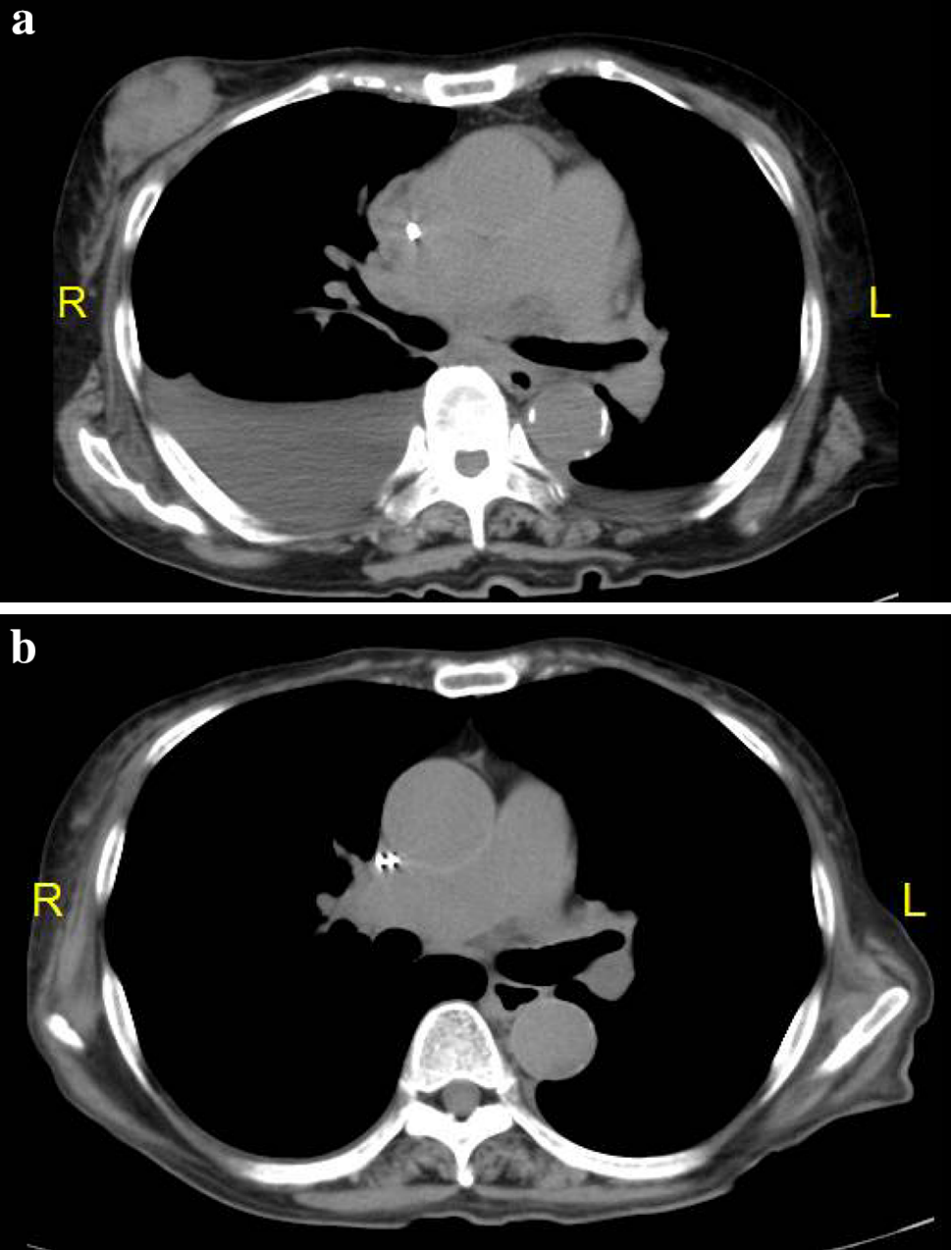
**a** Computed tomography showed a round mass, 4.8 × 2.6 × 5 cm, in the outer *upper* quadrant of the *right* breast with pleural effusions in both lungs before treatment. **b** The mass in the *right* breast and bilateral pleural effusions disappeared with nearly complete response after 6 cycles of CHOP chemotherapy

A 77-year-old woman presented to a city hospital with the chief complaint of a painless, palpable mass in the right breast; however, the results of fine needle aspiration cytology were inconclusive. One month later, computer tomography (CT) showed bilateral pleural effusions and she was moved to our university hospital. Routine physical examination revealed a 1.5-cm diameter, round mass in the upper outer quadrant of her right breast. A heterogenic mass was seen on ultrasonography (Fig. 1b), but nothing was seen on a diagnostic mammogram (Fig. 1a). Her medical history included a 15-year history of hypertension, well controlled with medication. Results of a staging chest X-ray film, a complete blood count, and liver function tests were normal, except for a high IL-2R of 1803 U/ml. Magnetic resonance imaging (MRI) revealed a 2.2-cm diameter mass in the right breast, showing a malignant imaging pattern in dynamic study, but no metastasis in the bilateral axillary lymph nodes (Fig. 2). Scintigraphy also showed increased gallium uptake in the left submandibular gland. Subsequent ultrasound-guided biopsy demonstrated atypical lymphocytes containing medium to large round nucleoli. Histologic analysis of the biopsy specimen was consistent with a peripheral T-cell lymphoma not otherwise specified and microscopic pathology revealed atypical lymphocytes containing medium to large round nucleoli and irregular nuclear shapes (Fig. 3a). Immunohistochemistry revealed CD2(+), CD3(+) (Fig. 3b), CD4(+), CD5(−), CD7(+), CD8(−), CD20(−) (Fig. 3c), CD25(−), CD30(+), CD56(−), bcl-2(−), EBV-ISH(−), TIA-I(−), and ATLA negative. Simultaneously, a 2.5-cm diameter mass was found in the left mandibular area. Fine needle aspiration was performed by an otolaryngologist and cytological examination revealed a malignant lymphoma with the features of PTCL-NOS. Although there was no evidence of metastasis in specimens of bone marrow biopsy, the cytology of the pleural effusion had shown the same involvement of malignant lymphoma. Even without associated lymph node involvement, we diagnosed Stage IV PTCL-NOS with three extralymphatic organs; namely the breast, mandibular gland, and pleural effusion, prior to the chemotherapy. On re-evaluating her initial breast MRI, the CT scan had shown the breast mass progressing rapidly with a significant increase in tumor size and in the pleural effusions (Fig. 4a) before treatment.

Based on the National Comprehensive Cancer Network (NCCN) guidelines, the patient was given systemic chemotherapy using the standard CHOP regimen (cyclophosphamide: 750 mg/m^2^; doxorubicin: 50 mg/m^2^; vincristine: 1.4 mg/m^2^; prednisone: 40 mg/m^2^) instead of surgical treatment. Six cycles of CHOP were administered at 21-day intervals over 4 months by the hematologist as follows: cyclophosphamide, doxorubicin and vincristine were given intravenously on day 1, with prednisone given orally 30 min prior to chemotherapy on day 1, then every 24 h on days 2–5.

CT scan showed a significant remission of the breast mass and pleural effusion in nearly complete response (Fig. 4b). However, the patient suffered some serious adverse effects during the chemotherapy, including grade 2 nausea, vomiting, and constipation, grade 3 neutropenia, and grade 2 heart disorder with palpitations and shortness of breath. Despite a dramatic response being achieved 6 months after beginning the CHOP therapy, it was discontinued because of heart failure, as recommended by the cardiologist. The disease relapsed in the third month after interrupting the CHOP, with a significant increase in the white blood cell count; however, no breast lump was detected at her last visit to the hospital. The patient did not wish to undergo any further aggressive therapy. She received palliative care until she died 17 months after the diagnosis.

## Discussion

PBL represents 0.2–1.5 % of breast malignancies [4–6], while T-cell lymphomas represent <15 % of all non-Hodgkin’s lymphomas (NHLs) [7]. Breast T-cell lymphomas are extremely rare and reported mainly as isolated cases. In fact, we found that only 17 cases were documented in Japan between 1983 and 2010.

The typical clinical symptom is one or multiple painless masses, similar to breast B-cell lymphoma and breast carcinoma. Enlarged ipsilateral axillary lymph nodes are reported in 13–50 % of PBL cases [8], although our patient did not present with this symptom. Interestingly, most cases of breast lymphoma have unexplained right side predominance, as in the present case. There are no pathognomonic mammographic features for breast lymphoma in general, and sometimes these lesions are only detected by ultrasound [9].

Although a palpable mass in the left submandibular gland was found simultaneously in our patient, the gastrointestinal tract and nasopharyngeal topography are the most frequent sites of involvement. When breast involvement is the presenting manifestation, it usually occurs in the context of other systemic disease. Involvement of the breast by precursor T-lymphoblastic lymphoma/leukemia presents as a mass or as bilateral diffuse involvement [10]. Nevertheless, the subtype of adult T-cell lymphoma/leukemia (ATLL) is most frequent in Japan, whereas PTCL-NOS is the most common type in North America and Europe, and natural killer/T-cell lymphoma (NKTCL) and ATLL are common in Asia. Moreover, several reports document cases of T-cell breast lymphoma associated with a breast implant [11–17], with anaplastic large cell lymphoma (ALCL) being the most common lymphoma found proximal to the implant [12].

The pathogenesis of T-cell lymphoma in the breast is poorly understood because of its rarity. As shown in Table 1, histopathological analysis of the 17 cases of PBL revealed 5 cases of ATLL, 1 of PTCL-NOS, and the 12 remaining cases were difficult to classify or reclassify according to the criteria proposed by the World Health Organization classification of tumors of hematopoietic and lymphoid tissue. PTCL-NOSs are mainly nodal lymphomas, accounting for more than 50 % of all T-cell lymphomas in adults, whereas the present case was exceptionally unusual in that it was an extranodal, extracutaneous T-cell lymphoma with a well-defined mass, occurring in a setting of leukemic disease. To the best of our knowledge, this represents only the second case of primary PTCL-NOS of the breast in Japan. From several years ago, scientific societies have proposed guidelines to standardize therapeutic strategies. The NCCN offers different solutions based on histology and clinical stage according to the Ann Arbor Classification [1, 18, 19]. Resective biopsy and conservative surgery is sufficient for pathologic evaluation. In fact, the role of surgery for PBL should be limited to assisting in establishing a definitive diagnosis [1]. Our patient received only systemic chemotherapy without surgical resection, unlike in other reported cases. Little is documented about the progress of primary T-cell lymphoma of breast, and the clinical outcome of these patients is variable although most had an aggressive disease course. Uesato et al. [20] found that tumor diameter and lymphomatous dissemination to the axillary lymph nodes was associated with a poor prognosis, with only 10 % (1/10) 5-year overall survival (OS). In contrast, PTCL-NOS is usually aggressive and relapses are common [21–24]. Both clinical stage and the international prognostic index (IPI) score used for other non-Hodgkin lymphomas (NHLs) have demonstrated the correlation of the overall survival of these patients [25]. Patients with PTCL generally have a worse prognosis than patients with B cell NHL [21]. Accordingly, our patient also suffered relapse and survived for only 17 months after her diagnosis. New strategies for refractory PBL are warranted.

**Table 1:** Comparison of the cases of primary breast peripheral T-cell lymphoma in Japan

No	Cases	Year	Age/Sex	Site	Cell surface markers	Treatment	Follow-up
1	our case		61/F	Left	CD2(+), CD3(+), CD4(+), CD5(−), CD7(+), CD8(−), CD20(−), CD25(−), CD30(+), CD56(−), bcl-2(−), EBV-ISH(−), TIA-I(−), ATLA(−)	CHOP	DOD 17 months
2	Toyoizumi	1990	45/F	UN	UN	*Modified	UN
3	Kamakura	1990	69/F	Left	UN	Modified	UN
4	Yamamiya	1991	45/F	Left	CD45(+), CD45RO(+), CD20cy(−), pankeratin(−)	UN	UN
5	Yamahigashi	1992	44/F	UN	UN	Modified + CHOP, VP16, MTX	DOD 2 months
6	Kosaka	1992	69/F	Left	ATLA(+)	Patey + CHOP	AWD 18 months
7	Tanaka	1993	64/F	Both	CD20cy(−), CD43(+), CD45RO(+), CD45RA(−)	Modified + BEMP, PROSTETIN	DOD 14 months
8	Tujimoto	1993	58/F	Right	UN	Modified + CDDP, CBDCA, VP-16 + radiation	AWD 10 months
9	Saeki	1994	42/F	Left	CD(−), CD3(+), CD4(−), CD5(+), CD8(−), CD19(−), CD20(−), CD22(−), CD25(−)	Mastectomy + chemotherapy	UN
10	Sakota	1997	84/F	Left	CD20cy(−), CD45(+), CD45RO(+),AE1/AE3(−), keratin(−), ATLA(+)	Lumpectomy	DOD 15 months
11	Mukaigawa	1998	68/F	Right	CD20cy(−), CD43(+), CD45RO(+), ATLA(−)	Auchincloss + CHOP, CPA**	AWD 6 months
12	Masatsuka	1998	41/F	Left	UN	Mastectomy	DOD 22 months
13	Shiiki	1999	45/F	UN	ATLA(+)	Modified + CHOP, Endoxan, ADR	DOD 19 months
14	Morozumi	1999	45/F	Left	CD20(−), CD45(+), CD45RO(+), AE1/AE3(+), ATLA(+)	Modified + CHOP, Bleo	DOD 19 months
15	Hasegawa	2000	48/F	Left	UN	breast-conserving surgery	DOD 72 months
16	Nomizu	2002	UN/F	UN	PTCL	UN	UN
17	Tanaka	2004	49/F	Left	CD20(−), CD45RO(+)	Modified + VEPA, VP16, CPA	DOD 32 months
18	Fujita	2004	45/F	Left	CD20cy(−), CD45RO(+), ATLA(+)	Breast-conserving surgery + CHOP, VP16	DOD 39 months
19	Murakami	2007	75/F	Right	CD3(+), CD45RO(+), ATLA(−)	Mastectomy + Epi-COP, G-IDEA	DOD 50 months

*UN* unknown, * *modified* modified radical mastectomy, *CHOP* cyclopholphamide, doxorubicin, vincristine, prednisolone, *VP16* etposide, *MTX* methotrexate, *BEMP* bleomycin, endoxan, methotrexate, prednisolone, *ADR* adriamycin, *Bleo* bleomycin, *VEPA* vincristine, cyclopholphamide, doxorubicin, prednisolone, *CPA* cyclopholphamide, *Epi*-*COP* epirubicin-based combination chemotherapy, *G-IDEA* G-CSF, ifosfamide, dexamethasone, etposide, cytarabine, *DOD* dead of disease, *AWD* alive with disease

Regarding the clinical approaches to primary peripheral T-cell lymphoma of the breast, women presenting with a breast mass should undergo a fine needle aspiration (FNA) or core needle biopsy (CNB). Moreover, women with abnormal imaging findings alone, such as abnormal calcification, should undergo biopsy guided by mammography, ultrasound, or breast MRI. Primary breast lymphoma, including PTCL-NOS, is a heterogeneous category and essentially a diagnosis of exclusion. In fact, the diagnostic agreement rate among expert pathologists is only about 75 % [26]. Although FNA is useful for distinguishing reactive B cell hyperplasias from clonal mature B cell neoplasms as an initial screening test [27–29], only an excisional biopsy of an intact node consistently obtains sufficient tissue for histologic, immunologic, molecular biologic assessment, and classification by experienced pathologists [30]. An accurate diagnosis of lymphoma based on FNA is not possible [31], which is probably why our patient came to our hospital without a definitive diagnosis.

In conclusion, we believe that patients with breast disease should undergo not only FNA, but also image-guided core biopsies to provide sufficient tissue for diagnosis. Complete evaluation, using an adequate tissue sample for accurate diagnosis, is most important to predict prognosis and design treatment for patients with primary peripheral T-cell lymphoma of the breast, including PTCL-NOS.
